# Charting health system reconstruction in post-war Liberia: a comparison of rural vs. remote healthcare utilization

**DOI:** 10.1186/s12913-016-1709-7

**Published:** 2016-09-07

**Authors:** Katherine Kentoffio, John D. Kraemer, Thomas Griffiths, Avi Kenny, Rajesh Panjabi, G. Andrew Sechler, Stephen Selinsky, Mark J. Siedner

**Affiliations:** 1 Last Mile Health, 1 Congress Street, Boston, MA 02114 USA; 2 Department of Medicine, Massachusetts General Hospital, 55 Fruit Street, Boston, MA 02114 USA; 3 Georgetown University Medical Center, 231 St. Mary’s Hall, 3700 Reservoir Road NW, Washington, DC 20057-1107 USA; 4 Last Mile Health, Hospital Road, Zwedru, Grand Gedeh Liberia; 5 Division of Global Health Equity, Brigham and Women’s Hospital, 75 Francis St, Boston, MA 02115 USA; 6 Harvard Medical School, Boston, MA USA

**Keywords:** Liberia, Rural health services, Health services accessibility, Maternal health, Child health

## Abstract

**Background:**

Despite a growing global emphasis on universal healthcare, access to basic primary care for remote populations in post-conflict countries remains a challenge. To better understand health sector recovery in post-conflict Liberia, this paper seeks to evaluate changes in utilization of health services among rural populations across a 5-year time span.

**Methods:**

We assessed trends in healthcare utilization among the national rural population using the Liberian Demographic and Health Survey (DHS) from 2007 and 2013. We compared these results to results obtained from a two-staged cluster survey in 2012 in the district of Konobo, Liberia, to assess for differential health utilization in an isolated, remote region. Our primary outcomes of interest were maternal and child health service care seeking and utilization.

**Results:**

Most child and maternal health indicators improved in the DHS rural sub-sample from 2007 to 2013. However, this progress was not reflected in the remote Konobo population. A lower proportion of women received 4+ antenatal care visits (AOR 0.28, *P* < 0.001) or any postnatal care (AOR 0.25, *P* <0.001) in Konobo as compared to the 2013 DHS. Similarly, a lower proportion of children received professional care for common childhood illnesses, including acute respiratory infection (9 % vs. 52 %, *P* < 0.001) or diarrhea (11 % vs. 46 %, *P* < 0.001).

**Conclusions:**

Our data suggest that, despite the demonstrable success of post-war rehabilitation in rural regions, particularly remote populations in Liberia remain at disproportionate risk for limited access to basic health services. As a renewed effort is placed on health systems reconstruction in the wake of the Ebola-epidemic, a specific focus on solutions to reach isolated populations will be necessary in order to ensure extension of coverage to remote regions such as Konobo.

**Electronic supplementary material:**

The online version of this article (doi:10.1186/s12913-016-1709-7) contains supplementary material, which is available to authorized users.

## Background

Over the last decade there has been a growing interest in provision of universally available primary care services in low-income countries, especially in those emerging from years of civil conflict. Investment in primary care can improve health outcomes, particularly among low socioeconomic groups, in a cost-effective manner [1–6]. This principle has been applied in Liberia following the end of a 13-year civil conflict in an effort to re-design fragmented, war crippled health systems along principles of equity and effectiveness [7–9].

Like other post-conflict states, the health system in Liberia is characterized by insufficient infrastructure, lack of appropriate qualified health personnel, and limited oversight capacity [10–13]. To address these varied challenges, Liberia’s post-war government chose to organize their reconstructive efforts around a Basic Package of Health Services (BPHS). The BPHS is a defined set of evidence-based, cost-effective interventions that are considered essential to improve the health of the population. By identifying high-priority services at the outset, the BPHS strategy aims to ensure that necessary interventions can be scaled in an organized and equitable manner across the entire health system, from rural clinics to district hospitals [11]. Several countries have taken this approach after the cessation of armed conflict in the last decade, including Afghanistan and South Sudan, with encouraging initial results [7, 14–16].

Following the conclusion of Liberia’s civil war in 2003, only 51 health facilities were functioning in the country, and only 10 % of the population was estimated to have access to basic healthcare [17]. The BPHS included plans to rebuild and staff health facilities, and established a minimum set of primary care interventions to be provided free of charge in all government clinics. These services focused particularly on maternal and child health, communicable disease, and mental heath [11]. Recognizing that a certain portion of the population would be unable to access facility-based services due to distance from available clinics, the BPHS also included a plan for the recruitment and training of community health volunteers (CHVs) to further expand certain services in rural regions. These services include linking women to health facilities for delivery and providing oral rehydration solution (ORS) and antibiotics to under-5 children with signs of diarrheal illness or pneumonia [18].

Implementation of the BPHS was rapid. According to the country’s first national clinic accreditation process, 80 % of government clinics were estimated to meet the minimum standards for delivery of the BPHS by 2010 [19–21]. The government therefore upgraded the BPHS to the Essential Package of Health Services (EPHS) in 2011, an expanded initiative that introduced a more comprehensive program for indicators (such as child health) that required increased attention [21].

While evidence suggests that this approach has been effective on a macro-level, there has been limited research into the success of the BPHS implementation among remote populations. This paper aims to analyze the availability of health services in Konobo District, Liberia, a remote region of the country, 5 years after the establishment of the BPHS—the period immediately before health systems were disrupted by the Ebola epidemic. Little data currently exist on this part of the country [22]. To better understand healthcare access in remote regions, we compare use of maternal and child health services in Konobo in 2012 with the rural sub-sample of the Liberian Demographic and Health Survey (DHS) conducted in 2007 (immediately after initiation of the BPHS) and in 2013 (the most recent survey) [23, 24]. Our goals are to 1) assess utilization of essential maternal and child health services among a particularly remote population; and 2) to compare utilization in this region with changes in health utilization more broadly across rural regions of the country.

## Methods

### Data sources

We used two data sources for this analysis. First, we use data from a maternal and child health survey conducted by Last Mile Health for program planning purposes in 2012 in Konobo. Details on the design and implementation of the survey are provided below. Permission to utilize the data for research purposes was granted by Last Mile Health. We compared data from Konobo with data drawn from the rural sub-sample of the Liberian DHS from 2007 and 2013. The DHS is a nation-wide, cross-sectional study of socio-demographic and health indicators, which seeks to provide representative information for assessing health trends within the population. The variables, data collection, quality assurance, and analysis have been described previously, and the full DHS datasets are made publically available [23, 24].

### Konobo study population

Konobo District (which as a health district contains the Konobo and Glio-Twarbo administrative districts) has a total population of 33,000 and a population density of less than 50 persons per square mile [25]. The 2007 DHS estimated that the region containing Konobo had the lowest asset index distribution in Liberia, with approximately half of residents living in the bottom quintile of wealth nationally, as measured by a household index of consumer goods and dwelling characteristics [23].

### Konobo survey sampling and data collection

The development, sampling methodology, and data collection of the Konobo survey have been previously described elsewhere [26]. Briefly, we assessed demographics and access to maternal healthcare for reproductively active women in Konobo. We also assessed episodes of illness and care seeking among respondents' children under-5 years old. For the Konobo population only, we summarized barriers to health care access, including distance and cost of transport to clinic. Participants were selected using two-stage representative cluster sampling, based on population data from the 2008 National Population and Housing Census [25]. We excluded Ziah Town, the district capital, because it met the Liberian definition of an urban area (i.e. population ≥ 2,000) [25]. An additional 25 villages were excluded because they had less than 20 households (*n* = 19), could only be reached on foot (*n* = 4) or could only be reached by canoe (*n* = 2). Together, residents from excluded villages comprised 15 % of Konobo’s rural population. Fever data was excluded from the final analysis due to very high reported prevalence, raising concern over question interpretation and result validity.

Both DHS surveys had broader sampling criteria than the Konobo Health Survey and initially approached all women aged 15–49 for inclusion. The Konobo survey limited data collection to the woman in each household > 17 years of age who had most recently given birth. In all three surveys, questions regarding maternal health were only asked regarding the most recent pregnancy among women who had given birth within the last 5 years. Questions about child health were only asked regarding each of the respondent’s own children < 5 years currently living in the household. To ensure comparability between the DHS and Konobo sampling frames, the following exclusions were applied prior to data analysis 1) we excluded women who had not given birth within the last 5 years and therefore were not eligible for the maternal health questions in all three surveys and 2) we excluded women under 17 years old from the DHS dataset and over 49 from the Konobo dataset, even if they had given birth in the last five years. Thus the final population for analysis across all three surveys includes reproductively active women who have given birth within the last 5 years and who were between the ages of 17 and 49 at the time data was collected.

### Statistical analyses

We first conducted descriptive analyses using standard statistical techniques: means and confidence intervals for normally distributed continuous variables, medians and inter-quartile ranges for other continuous variables, and proportions with confidence intervals for categorical variables. We incorporated sampling structure and weights and produced design-corrected standard errors using Taylor series linearization.

To make comparisons between survey years, we constructed a dataset that pooled data from the three surveys. We retained the complex sample design by keeping strata unique between surveys. Weights were rescaled so that relative weights for each observation were retained within each survey and each survey’s rural population contributed equally to the analysis. Our primary maternal health outcomes of interest were: 1) one or more antenatal care visits with a skilled provider (1+ ANC), 2) four or more ANC visits, at least one from a skilled provider (4+ ANC), 3) delivery in a health facility, and 4) post-natal care (PNC) from a skilled provider within 24 h of delivery. A skilled provider was defined as a doctor, nurse/midwife or physician’s assistant in the DHS and as a doctor, nurse or physician’s assistant in the Konobo survey. The combined term nurse/midwife was not used in Konobo as this was found to lack clarity in pilot testing. Traditional midwives were not considered “skilled providers,” as we have limited data on how these practitioners are trained in many regions of the country. Our primary child health outcomes were 1) prevalence of acute respiratory illness (ARI), defined as cough, difficulty breathing, and chest involvement within the past 2 weeks; 2) prevalence of diarrhea within the past 2 weeks, 3) receipt of care during an ARI or diarrheal episode from a health facility, and 4) receipt of care for either condition from any provider (including informal and traditional providers such as pharmacists and “tablet men,” individuals who sell common pharmaceuticals of unclear provenance in marketplaces).

We fitted multivariable logistic regression models, using sampling weights and design-corrected standard errors, for each outcome of interest. We adjusted for potential confounders that have been hypothesized to influence care utilization, but which are outside the causal pathway for public health sector improvement. The maternal health model included variables for maternal age, marital status, age at first birth, education and birth order (defined as the first, second or third or greater birth). For the child health model, we also included child age and gender in addition to the maternal health variables listed above. For each outcome of interest, we then calculated predicted adjusted probabilities by survey, using post-regression marginal effects with other model covariates held at their mean values. Data analysis was performed with Stata Version 13.0 (Statacorp, College Station, Texas).

## Results

### Respondent characteristics

Five hundred fifty-five women in total completed the 2012 Konobo survey, of whom 428 (77.1 %) had been pregnant in the last 5 years (Table 1). Respondents reported a total of 556 under-five children currently living in their households. The median age of respondents was 30 years (IQR 26–38 years), with a median age at first birth of 17 (IQR 16–19). In comparison to women who completed the Konobo survey, women who completed either DHS were of similar age, were more likely to have never been to school, and were more likely to live within 2 h walk from a health facility (data not available for 2007). There was no meaningful difference in median age at first birth between groups. Women who completed the Konobo survey reported more lifetime births (4.40, 95 % CI 4.09–4.76) than women in the 2007 DHS survey (3.89, CI 3.78–4.01).

**Table 1:** Demographic Characteristics of Respondents

Variable	2007 DHS (Rural)	2013 DHS (Rural)	Konobo
Sample Size			
Women	3,634	5,061	555
Households	4,218	5,883	555
Median age, years (IQR)	30 (24–39)	30 (23–39)	30 (26–38)
Median age, years (IQR)			
17–19	8.8 (7.7–10.1)	11.2 (10.2–12.3)	3.8 (2.5–5.8)
20–24	19.7 (18.2–21.3)	17.5 (16.2–18.7)	16.4 (13.3–20.0)
25–29	17.4 (15.8–19.2)	18.5 (17.2–19.9)	22.4 (18.4–27.1)
30–34	14.7 (13.2–16.4)	15.3 (13.9–16.8)	21.0 (17.6–24.9)
35–39	15.5 (14.1–17.0)	14.5 (13.3–15.8)	14.2 (11.3–17.6)
40–44	11.1 (10.0–12.3)	11.7 (10.7–12.9)	13.2 (10.7–16.3)
45–49	12.8 (11.3–14.4)	11.4 (10.2–12.7)	9.0 (6.7–11.9)
Mean household size, persons (95 % CI)	4.96 (4.80–5.11)	5.0 (4.86–5.15)	5.99 (5.61–6.37)
Education, % (95 % CI)			
None	58.2 (53.8–62.4)	53.7 (50.9–56.6)	27.6 (23.2–32.3)
Primary	31.8 (28.7–35.1)	31.4 (29.5–33.3)	50.0 (44.9–55.0_
Secondary or higher	10.0 (8.11–12.2)	14.9 (13.2–16.9)	22.5 (16.6–29.7)
Median age at first birth for women 20–49, years (IQR)	18.0 (16–21)	18 (16–20)	17 (16–19)
Median children ever born, persons (IQR)	3 (1–5)	3 (1–6)	4 (2–6)
Children ever-born, persons, mean (95 % CI)	3.89 (3.78–4.01)	4.08 (3.94–4.21)	4.40 (4.09–4.76)
17–19	0.65 (0.53–0.78)	0.69 (0.63–0.74)	1.32 (1.06–1.59)
20–24	1.62 (1.51–1.72)	1.86 (1.78–1.95)	2.32 (2.02–2.62)
25–29	2.97 (2.81–3.12)	3.22 (3.11–3.33)	3.09 (2.73–3.44)
30–34	4.12 (3.94–4.32)	4.59 (4.43–4.76)	4.04 (3.56–4.53)
35–39	5.39 (5.11–5.67)	5.71 (5.53–5.89)	5.20 (4.79–6.44)
40–44	6.33 (6.02–6.65)	6.24 (5.92–6.55)	6.86 (6.17–7.55)
45–49	6.70 (6.39–7.01)	7.18 (6.82–7.54)	8.11 (7.28–8.94)
Pregnant in last 5 years, % (95 % CI)	67.9 (65.6–70.1)	66.4 (64.7–68.1)	77.1 (72.7–80.9)
Walking time to nearest facility, % (95 % CI)			
60 min or less	Data not available	40.6 (33.7–47.8)	10.8 (5.0–21.9)
61–120 min	Data not available	27.5 (23.3–32.0)	16.0 (8.8–27.4)
> 120 min	Data not available	32.0 (26.6–37.8)	73.2 (59.5–83.5)

### Access to maternal healthcare

**Fig. 1:**
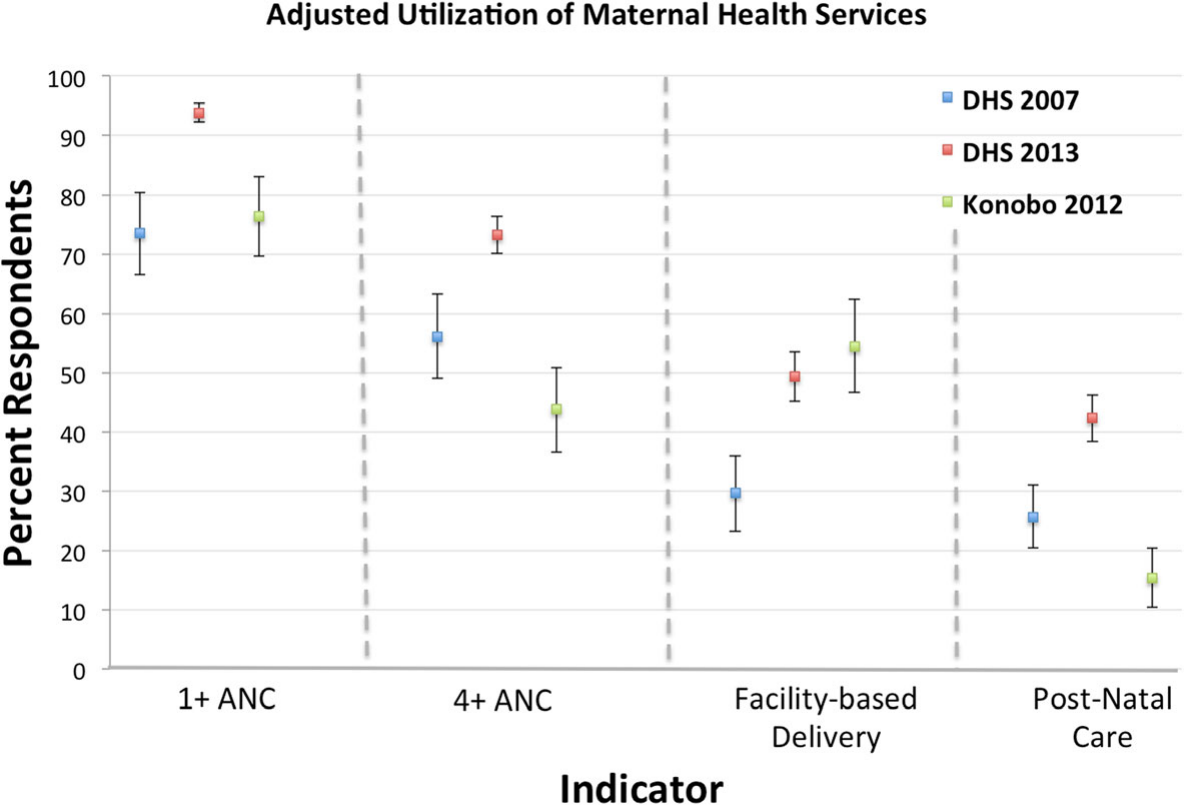
Maternal Health Services Utilization: Adjusted mean and 95 % confidence intervals for receipt of maternal health services among respondents to the Konobo survey as compared to the rural subsection of DHS 2007 and DHS 2013

All four maternal health indicators (any ANC, 4+ ANC visits, delivery in a health facility, and PNC) demonstrated significant improvement between 2007 and 2013 in the rural DHS sub-sample (Table 2). These improvements persisted after adjustment for potential cofounders (Fig. 1, Additional file 1: Table S1). However, residents in Konobo in 2012 reported rates of utilization significantly below those documented in 2013 for most indicators.

**Table 2:** Receipt of Maternal and Child Health Services (Unadjusted)

Variable	2007 DHS (Rural)	2013 DHS (Rural)	Konobo
Sample Size (N)	2447 births	3448 births	430 births
% Population, (95 % CI)			
Any ANC visit from a skilled provider	71.8 (64.3–78.2)	93.4 (91.7–94.9)	76.5 (70.5–81.7)
4+ ANC visits (1+ from skilled provider)	55.2 (48.3–61.9)	72.9 (69.9–75.8)	44.0 (38.0–50.2)
Delivery in a health facility	28.8 (23.1–35.3)	48.7 (44.7–52.6)	55.1 (48.0–62.0)
PNC within 24 h of delivery from a skilled provider	24.9 (20.1–30.5)	41.7 (37.9–45.6)	17.0 (12.9–22.1)
Sample Size (N)	3420 living children	4792 living children	556 living children
% Population, (95 % CI)			
Children with ARI in last 2 weeks	9.8 (8.0–12.0)	7.9 (6.8–9.2)	21.5 (17.1–26.8)
Children evaluated for ARI by any provider	84.1 (77.9–88.8)	71.2 (63.3–78.0)	76.1 (66.3–83.7)
Children evaluated for ARI by a skilled provider	62.8 (55.7–69.3)	51.6 (44.3–58.8)	8.9 (4.6–16.5)
Children with diarrhea in last 2 weeks	21.2 (18.6–24.0)	24.8 (22.7–27.0)	46.7 (41.4–52.1)
Children evaluated for diarrhea by any provider	80.4 (75.3–84.6)	73.5 (69.0–77.5)	64.1 (55.5–71.9)
Children evaluated for diarrhea by a skilled provider	49.2 (41.8–56.6)	46.1 (40.8–51.5)	11.4 (6.5–19.3)

Between 2007 and 2013, rates of attendance of any ANC visit increased significantly among the rural DHS subset from 72 to 93 % of women (AOR 0.18, *P* < 0.001). Among Konobo respondents, crude rates of 1+ ANC (77 %) were significantly below DHS 2013 (93 %). Adjusted odds ratios for receiving 1+ ANC for Konobo versus DHS 2013 was 0.21 (CI 0.13–0.34, *P* < 0.001). Odds of receiving 4 + ANC visits were also significantly lower in Konobo vs. in DHS 2013 (Table 3).

**Table 3:** Odds of obtaining maternal and child health services compared to the 2013 rural DHS

Variable	2007 Rural DHS Sub-Sample versus 2013 Rural DHS Sub-Sample		Konobo District versus 2013 Rural DHS Sub-Sample	
	AOR (95 % CI)	*P*-value	AOR (95 % CI)	*P*-value
Any ANC visit from a skilled provider	0.18 (0.12–0.28)	<0.001	0.21 (0.13–0.34)	<0.001
4+ ANC visits (1+ from skilled provider)	0.47 (0.34–0.64)	<0.001	0.28 (0.20–0.41)	<0.001
Delivery in a health facility	0.43 (0.31–0.61)	<0.001	1.23 (0.84–1.79)	0.286
PNC within 24 h of delivery from a skilled provider	0.47 (0.34–0.65)	<0.001	0.25 (0.16–0.38)	<0.001
Children with ARI	1.29 (0.97–1.71)	0.078	2.32 (1.59–3.38)	<0.001
Children evaluated for ARI by any provider	2.45 (1.36–4.40)	0.003	0.91 (0.39–2.15)	0.831
Children evaluated for ARI by a skilled provider	1.55 (1.01–2.39)	0.046	0.04 (0.02–0.09)	<0.001
Children with diarrhea	0.81 (0.66–0.99)	0.042	3.24 (2.35–4.47)	<0.001
Children evaluated for diarrhea by any provider	1.50 (1.02–2.22)	0.040	0.72 (0.43–1.21)	0.215
Children evaluated for diarrhea by a skilled provider	1.15 (0.79–1.68)	0.451	0.08 (0.04–0.15)	<0.001

As with ANC, rate of delivery in a health facility increased in the rural DHS between 2007 and 2013 from 29 % of women to 49 % of women (AOR 0.43 for 2007 compared to 2013, *P* < 0.001). However, in contrast to utilization of ANC, there was no significant difference in utilization of delivery in a health facility in Konobo (55 %) as compared to rural averages in 2013 (49 %, AOR Konobo versus 2013 = 1.23, *P* = 0.29).

Finally, while receipt of PNC services within 24 h of delivery increased significantly in the DHS population between 2007 (25 %) and 2013 (42 %, AOR 0.47, CI 0.34–0.65, *P* < 0.001), the rates in Konobo in 2012 (17 %) also fell significantly below the 2013 DHS estimates (AOR Konobo versus 2013 = 0.25, CI 0.16–0.38, *P* < 0.001).

### Access to child healthcare

**Fig. 2:**
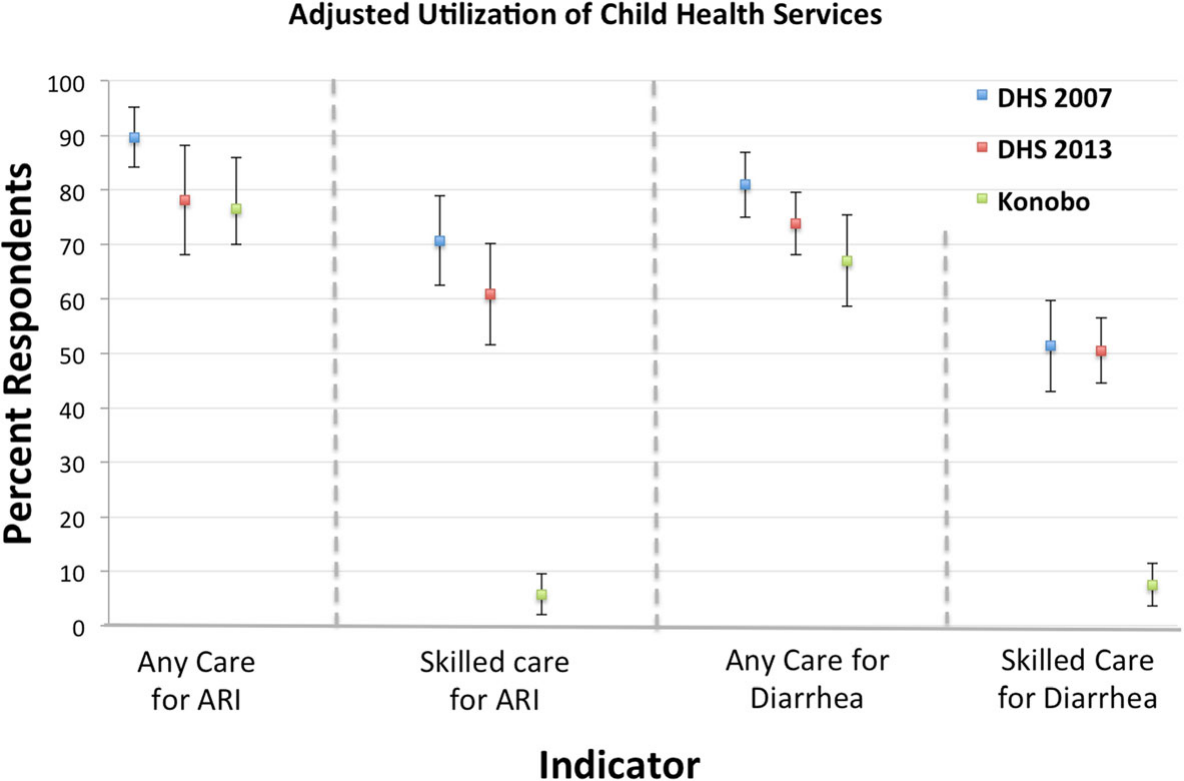
Child Health Services Utilization: Adjusted mean and 95 % confidence interval for prevalence of childhood illness and receipt of care among respondents to the Konobo survey as compared to the rural subsection of DHS 2007 and DHS 2013

There was no significant difference in the prevalence of ARI in the 2007 DHS (10 %) as compared to the 2013 DHS (8 %, AOR 1.29, CI 0.97–1.71, *P* = 0.078). Prevalence of ARI symptoms in Konobo was significantly higher (22 %), (Konobo versus DHS 2013, AOR 2.32, CI 1.59–3.38, *P* < 0.001). Similar findings were noted in the prevalence of diarrhea (Fig. 2).

Rates of health care seeking with a skilled provider for these conditions were similar in 2007 and 2013 in the rural DHS (ARI 63 and 51 %, AOR 1.55, CI 1.01–2.39, *P* = 0.046); diarrhea 49 and 46 %, AOR 1.15, CI 0.79–1.68, *P* = 0.451). Yet, rates of care seeking from a skilled provider for both ARI and diarrhea were much lower in Konobo (9 and 11 %, respectively, ARI care seeking in Konobo versus 2013 AOR 0.04, CI 0.02–0.09, *P* < 0.001; for diarrhea care seeking in Konobo versus 2013, AOR 0.08, CI 0.04–0.15, *P* < 0.001). When we expanded the definition of a provider to include any provider (including informal and traditional providers), the observed differences in care seeking behavior between Konobo and the 2013 DHS were no longer significant in both models (ARI care AOR = 0.91, *P* = 0.831; diarrhea care AOR = 0.72, *P* = 0.215).

### Barriers to healthcare access in the Konobo population

Seventy-three percent of women in the Konobo survey (CI 60–84 %) reported taking greater than 2 h to walk to the nearest clinic (versus 32 % of women in the 2013 rural DHS, CI 27–38 %). In the Konobo survey population, the average fee for a paid motorbike to clinic was $7.87 USD. The median distance by road from home to clinic as measured by global positioning systems was 28.9 km (range 3.5–50.2 km).

## Discussion

The analysis of DHS data demonstrated measurable gains across a range of maternal health services for the aggregate rural population in Liberia between 2007 and 2013. These improvements are likely related, at least in part, to heath reconstruction efforts under the BPHS. However, child health outcomes have notably lagged behind improvements in maternal health care. In our analysis, initial rates of care utilization were low. There was a borderline significant decline in skilled care for children with ARI between 2007–2013, and there was no measureable change in utilization for children with diarrhea in the same period.

In contrast to the improvements seen in maternal health among the rural subset as a whole, our analysis of health access in Konobo District indicates that marked disparities exist in this remote region. The most significant deficiencies were noted in prenatal care, postnatal care, and management of childhood illness, where utilization in Konobo in 2012 was significantly below Liberian rural estimates from 2013, despite the preceding 5 years of BPHS implementation. While our analysis is limited to only one specific region of Liberia, it is likely comparable to other similarly remote areas of the country [9, 27].

Interestingly, the one place where we did not identify a disparity was in delivery in a health facility, where we found no significant difference between Konobo and DHS 2013. While this may be due to health behaviors specific to women in Konobo, it is also worth noting that facility-based delivery has been a priority for the Liberian Ministry of Health, and that improvements in this metric have been strong in the majority of counties according to the DHS. Furthermore, Grand Gedeh, the county containing Konobo, had a disproportionate increase in the number of skilled birth attendants per 10,000 persons as compared the national average in the period of 2010 to 2015, which may have improved rates of facility based delivery in Konobo [27, 28].

The implementation of the BPHS has generally been considered successful in Liberia [21]. However, our analysis of both the DHS data broadly, and of Konobo is particular, indicates that there is still significant progress to be made in order to ensure equitable access to all recommended maternal and child health services across the rural population as a whole. Furthermore, the direct and indirect effects of the 2014 Ebola epidemic are likely to exacerbate already existing challenges in healthcare delivery [29]. Previous research has demonstrated that the Ebola epidemic has put significant strain on health infrastructure through loss of health workers, reallocation of already scarce program resources, and decreased trust in the health system [30–32]. These challenges have been documented in many settings across Liberia, including those areas where viral transmission was less intense, such as the Southeast region containing Konobo [33].

To plan health system reconstruction in the wake of Ebola, a thorough conceptualization of facilitators and barriers to health for rural citizens will be essential. In our population, women most often reported barriers to care related to issues of distance and transportation. The median distance to the health facility was long (29 km by road as measured by GPS) and the estimated cost of transportation was high (nearly $8 USD), despite the fact that nearly two in three Liberians earn less than $1 USD per day [34]. Given the large proportion of the population that lives > 5 km from a health clinic (estimated at 28 % nationally), facility-based systems of health delivery will be unlikely to provide adequate coverage in many rural regions [28]. The establishment of mobile health clinics and a paid cadre of community health workers has demonstrated efficacy at improving front-line healthcare and strengthening referral services in other areas of the world, where long distances to health facilities are a major concern for stakeholders [35–39]. However, there are many barriers to this strategy within the Liberian context. Previous research in a multitude of settings has demonstrated that the efficacy of community health workers is strongly tied to a number of intersecting incentives, including provision of high-quality training, adequate supervision, and integration into established health networks [40, 41]. However, a 2014 analysis found only patchy community participation in CHV selection under the BPHS with widely varying capacity to implement the range of maternal and child health interventions mandated for rural regions. Supervision and support were generally found to be inadequate [18]. A subsequent post-Ebola analysis found most existing CHVs to be operating in partnership with non-profit governmental partners rather than directly within the national health system [42]. Granular data on the distribution and capacity of CHVs by county or district are sparse. Significant work remains to be done if CHVs are to be the primary providers of the EPHS for communities living at significant distances from health clinics, including establishing clear protocols for CHV responsibilities and ensuring their equitable distribution throughout communities.

Finally, it is important to recognize that although access is a major issue, healthcare quality must also be rigorously monitored and addressed if health outcomes are to be improved [16, 42]. The first step to true quality improvement for remote areas will require data collection methodologies that allow for the accurate representation of health metrics from these regions. Furthermore, in remote districts where operating expenses related to providing health care are higher, new metrics that incorporate population density should be analyzed as an alternative to simple per capita allocations when creating health budgets. More work is needed to determine how best to link improvements in access and quality such that an effective and equitable health system can ultimately be realized.

### Limitations

Our study has important limitations. First, our population of interest was limited to reproductively active women within the catchment area of a single district clinic, which affects the generalizability of our results. However, since fertility rates are high in Liberia, and since both our study and the DHS limit data collection on maternal and child health indicators to women who have given birth within the last 5 years, our respondents are likely to be quite similar demographically to those of the larger DHS sample. This is evidenced by the similar characteristics noted between the survey populations. Second, while we compared the Konobo sample in 2012 to nationally representative samples from the DHS in 2007 and 2013, we do not have data to assess trends in Konobo itself. As such, we can only make inferences about how changes in Konobo may compare to rural areas of the country over the last few years.

## Conclusions

Despite national progress in improving access to maternal and child health services in rural areas, deep deficiencies in access to basic health infrastructure exist in Konobo. The extent of the deficiencies spanned many aspects of maternal and child health care. A renewed focus on universal access to high quality primary care in the most difficult to reach populations is essential to erase the health deficits observed in remote areas of Liberia.

## Additional file

Additional file 1: Table S1. Receipt of Maternal and Child Health Services (Adjusted): Percent of the population receiving maternal and child health services in the rural subsection of DHS 2007, DHS 2013 and the Konobo survey, with 95 % confidence intervals. (DOCX 96 kb)

## Abbreviations

ANC: Antenatal care
AOR: Adjusted odds ratio
ARI: Acute respiratory illness
BPHS: Basic package of health services
CHV: Community health volunteer
CI: Confidence interval (95 %)
DHS: Demographic and health survey
EPHS: Essential package of health services
GPS: Global positioning system
IQU: Interquartile range
MOH: Ministry of health
ORS: Oral rehydration solution
PNC: Post natal care
USD: United States dollar
