# Supplementary material for: Charting health system reconstruction in post-war Liberia: a comparison of rural vs. remote healthcare utilization
Source: BMC Health Serv Res. 2016 Sep 7;16(1):478. doi: 10.1186/s12913-016-1709-7 (PMC5015243; doi:10.1186/s12913-016-1709-7)
Supplement: Additional file 1: Table S1. — Receipt of Maternal and Child Health Services (Adjusted): Percent of the population receiving maternal and child health services in the rural subsection of DHS 2007, DHS 2013 and the Konobo survey, with 95 % confidence intervals. (DOCX 96 kb) [file 12913_2016_1709_MOESM1_ESM.docx]

| Variable | 2007 DHS (Rural) | 2013 DHS (Rural) | Konobo |
| --- | --- | --- | --- |
| Sample Size (N) | 2447 births | 3448 births | 430 births |
| % Population, (95% CI) |  |  |  |
| Any ANC visit from a skilled provider | 73.5 (66.5 – 80.4) | 93.8 (92.3 – 95.4) | 76.3 (69.6 – 83.0) |
| 4+ ANC visits (1+ from skilled provider) | 56.1 (49.0 – 63.2) | 73.2 (70.1 – 76.3) | 43.8 (36.6 – 50.9) |
| Delivery in a health facility | 29.7 (23.3 – 36.0) | 49.4 (45.2 – 53.5) | 54.5 (46.7 – 62.3) |
| PNC within 24 hours of delivery from a skilled provider | 25.7 (20.5 – 31.0) | 42.4 (38.4 – 46.3) | 15.4 (10.4 – 20.4) |
| Sample Size (N) | 3420 living children | 4792 living children | 556 living children |
| % Population, (95% CI) |  |  |  |
| Children with ARI in last two weeks | 10.4 (8.2 – 12.7) | 8.3 (7.0 – 9.6) | 17.4 (12.8 – 22.0) |
| Children evaluated for ARI by any provider | 89.7 (84.2 – 95.3) | 78.1 (68.1 – 88.2) | 76.5 (70.0 – 86.0) |
| Children evaluated for ARI by a skilled provider | 70.7 (62.4 – 79.0) | 60.9 (51.6 – 70.2) | 5.7 (2.0 – 9.5) |
| Children with diarrhea in last two weeks | 19.7 (16.8 – 22.5) | 23.2 (20.8 – 25.5) | 49.5 (43.0 – 55.9) |
| Children evaluated for diarrhea by any provider | 81.0 (75.0 – 86.9) | 73.9 (68.1 – 79.6) | 67.0 (58.7 – 75.4) |
| Children evaluated for diarrhea by a skilled provider | 54.1 (45.7 – 62.5) | 50.5 (44.5 – 56.5) | 7.5 (3.6 – 11.4) |

**Supplemental Table: Receipt of Maternal and Child Health Services (Adjusted)**
